# High Heterogeneity of *Escherichia coli* Sequence Types Harbouring ESBL/AmpC Genes on IncI1 Plasmids in the Colombian Poultry Chain

**DOI:** 10.1371/journal.pone.0170777

**Published:** 2017-01-26

**Authors:** Luis Ricardo Castellanos, Pilar Donado-Godoy, Maribel León, Viviana Clavijo, Alejandra Arevalo, Johan F. Bernal, Arjen J. Timmerman, Dik J. Mevius, Jaap A. Wagenaar, Joost Hordijk

**Affiliations:** 1 Department of Infectious Diseases and Immunology, Faculty of Veterinary Medicine, Utrecht University, Utrecht, the Netherlands; 2 Colombian Integrated Program for Antimicrobial Resistance Surveillance – Coipars, Corporación Colombiana de Investigación Agropecuaria - Corpoica, Cundinamarca, Colombia; 3 Instituto Colombiano Agropecuario - ICA, Bogotá, Colombia; 4 Department of Biological Sciences, Los Andes University, Bogotá, Colombia; 5 Wageningen Bioveterinary Research, Lelystad, the Netherlands; Ross University School of Veterinary Medicine, SAINT KITTS AND NEVIS

## Abstract

**Background:**

*Escherichia coli* producing ESBL/AmpC enzymes are unwanted in animal production chains as they may pose a risk to human and animal health. Molecular characterization of plasmids and strains carrying genes that encode these enzymes is essential to understand their local and global spread.

**Objectives:**

To investigate the diversity of genes, plasmids and strains in ESBL/AmpC-producing *E*. *coli* from the Colombian poultry chain isolated within the Colombian Integrated Program for Antimicrobial Resistance Surveillance (Coipars).

**Methods:**

A total of 541 non-clinical *E*. *coli* strains from epidemiologically independent samples and randomly isolated between 2008 and 2013 within the Coipars program were tested for antimicrobial susceptibility. Poultry isolates resistant to cefotaxime (MIC ≥ 4 mg/L) were screened for ESBL/AmpC genes including *bla*_CTX-M_, *bla*_SHV_, *bla*_TEM_, *bla*_CMY_ and *bla*_OXA_. Plasmid and strain characterization was performed for a selection of the ESBL/AmpC-producing isolates. Plasmids were purified and transformed into *E*. *coli* DH10B cells or transferred by conjugation to *E*. *coli* W3110. When applicable, PCR Based Replicon Typing (PBRT), plasmid Multi Locus Sequence Typing (pMLST), plasmid Double Locus Sequence Typing (pDLST) and/or plasmid Replicon Sequence Typing (pRST) was performed on resulting transformants and conjugants. Multi Locus Sequence Typing (MLST) was used for strain characterization.

**Results:**

In total, 132 of 541 isolates were resistant to cefotaxime and 122 were found to carry ESBL/AmpC genes. Ninety-two harboured *bla*_CMY-2_ (75%), fourteen *bla*_SHV-12_ (11%), three *bla*_SHV-5_ (2%), five *bla*_CTX-M-2_ (4%), one *bla*_CTX-M-15_ (1%), one *bla*_CTX-M-8_ (1%), four a combination of *bla*_CMY-2_ and *bla*_SHV-12_ (4%) and two a combination of *bla*_CMY-2_ and *bla*_SHV-5_ (2%). A selection of 39 ESBL/AmpC-producing isolates was characterized at the plasmid and strain level. ESBL/AmpC genes from 36 isolates were transferable by transformation or conjugation of which 22 were located on IncI1 plasmids. These IncI1 plasmids harboured predominantly *bla*_CMY-2_ (16/22), and to a lesser extend *bla*_SHV-12_ (5/22) and *bla*_CTX-M-8_ (1/22). Other plasmid families associated with ESBL/AmpC-genes were IncK (4/33), IncHI2 (3/33), IncA/C (2/33), IncΒ/O (1/33) and a non-typeable replicon (1/33). Subtyping of IncI1 and IncHI2 demonstrated IncI1/ST12 was predominantly associated with *bla*_CMY-2_ (12/16) and IncHI2/ST7 with *bla*_CTX-M-2_ (2/3). Finally, 31 different STs were detected among the 39 selected isolates.

**Conclusions:**

Resistance to extended spectrum cephalosporins in *E*. *coli* from Colombian poultry is mainly caused by *bla*_CMY-2_ and *bla*_SHV-12_. The high diversity of strain Sequence Types and the dissemination of homogeneous IncI1/ST12 plasmids suggest that spread of the resistance is mainly mediated by horizontal gene transfer.

## Introduction

Infections caused by isolates of *Escherichia coli* resistant to extended spectrum cephalosporins (ESC), can result in antimicrobial treatment failure and increased health expenditures in humans [1–3], livestock [4] and companion animals [5]. Isolates resistant to ESC produce enzymes capable of hydrolyzing the β-lactam ring of these drugs, and exhibit reduced susceptibility to third generation cephalosporins and monobactams. These ESC hydrolyzing enzymes are mostly plasmid mediated and they include Extended Spectrum Beta-Lactamases (ESBLs), and AmpC beta-lactamases [6,7]. ESBL/AmpC-producing *E*. *coli* have been isolated from humans as well as from livestock, animal meat products, companion animals [8], and vegetables [9]. ESBL/AmpC-producing *E*. *coli* have been frequently reported in poultry and therefore poultry production and poultry meat are considered a reservoir of ESBL/AmpC-producing *E*. *coli* potentially causing infections in humans [10–15].

Previous studies have compared the occurrence and characteristics of genes, plasmids and strains of ESBL/AmpC-producing *E*. *coli* from humans, poultry and poultry products [11,16,17]. Some of these studies have found genetic relatedness of strains, genes and/or plasmids, suggesting transmission of ESBL/AmpC-producing *E*. *coli* between poultry and humans. Some studies suggest the transmission of bacterial strains, with plasmids and genes through clonal spread [12,16] while others suggest the transmission of plasmids conferring the resistance through horizontal gene transfer between different *E*. *coli* strains [13,17]. Whether this transmission happens by means of clonal spread or dissemination of mobile genetic elements, likely corresponds to different conditions within the analysed geographical areas [18].

In Colombia, available literature from human clinical isolates shows that the most frequent ESBL- gene variants are *bla*_CTX-M-12_, *bla*_CTX-M-15_, *bla*_SHV-12_ and *bla*_SHV-5_ [19–24]. More recently, some of these studies included AmpC-genes on their genotypic screening, and demonstrated the association of *bla*_CMY-2_ to community onset infections [22,23]. Nevertheless, reports that describe the genetic determinants of ESBL/AmpC-producing *E*. *coli* from Colombian poultry are not available. This information is essential to know whether a proportion of the mobile genetic elements harbouring ESBL/AmpC—producing *E*.*coli*, in particular plasmids, identified in isolates from poultry and humans are genetically related. Therefore, the aim of this study was to characterize the genes, plasmids and strains of a collection of ESBL/AmpC-producing *E*. *coli* from baseline studies in the Colombian poultry chain using sequence-based characterization that enables comparisons regardless of time, place and source of isolates.

## Materials and Methods

### Origin of samples and isolation of *E*. *coli*

Non-clinical samples were obtained from three different sources from the Colombian poultry production chain: i) broiler farms ii) broilers at slaughter and iii) raw chicken meat at retail. The samples from farms (n = 1097), slaughterhouses (n = 1566), and retail (n = 1203) were taken as part of a pilot project for the establishment of the Colombian Integrated Program for Antimicrobial Resistance Surveillance (Coipars) [25]. The samples were collected between 2008 and 2013 and originated from i) feces, drag swabs and cloacal swabs from broiler farms ii) cecal content and carcass rinse from slaughterhouses iii) chicken thighs (homogenized with stomacher method) from independent stores and a large retail distribution centre. Carcass rinse samples were obtained from independent stores and supermarkets. Three out of 32 departments of Colombia (that is, provinces) responsible for more than 65% of Colombian chicken production, were represented at the farm level [26]. At the slaughterhouse and retail level, samples were collected from the most populated departments, 18/32 and 23/32, respectively. After collection, samples were stored in insulated containers at 4°C and transported to the laboratory within 24 hours.

Isolates were recovered from samples from the three sources using the methodology previously described by the Canadian Integrated Program for Antimicrobial Resistance Surveillance (CIPARS) [27]. Briefly, samples were mixed in a 1:10 ratio (w/v) with buffered peptone water (BD, USA) and one loop of the mixture was streaked on a MacConkey agar plate (BD, USA) and incubated overnight at 35°C. For chicken thighs samples, 50 ml of EC broth (7% w/v) (BD, USA) was added to the mixture and incubated overnight at 45°C for 24 hours. Next, one loop of the mixture was streaked on a MacConkey agar plate (BD, USA) and incubated overnight at 35°C.

From MacConkey agar plates, one typical lactose-fermenting colony was further plated on BHI agar and incubated overnight at 35°C. One colony of the pure culture was screened with Sulphur Indole and Motility medium (SIM) (Difco, USA). Subsequently, presumptive *E*. *coli* strains (negative H_2_S production and positive tryptophan degradation) were stored at -80°C on sterile Skim Milk (20% w/w) (Difco, USA).

### Analyzed strains, antimicrobial resistance screening and species confirmation

From the stored isolates from farms and slaughterhouses, a convenient selection was made. Given the country-wide scope of an integrated surveillance program like Coipars, and the absence of previous reports of ESC resistance from poultry in Colombia, our strategy aimed to represent as much levels, years of production and departments as possible. A sum of 155 and 182 isolates representing 14% and 12% of the total number of samples, respectively, was made in a way that at least one isolate originated from every sampled department. In order to avoid overrepresentation of genes, plasmids and strains due to sample duplicity, repeated isolates from the same production flocks (that is, epidemiologically related isolates) were excluded. In contrast to samples from farms and slaughter, each isolate from retail originated from epidemiologically unrelated flocks and were therefore all included to assess the level of exposure to retail consumers and widen the comparisons with isolates from humans in Colombia. Based on this selection, 541 epidemiologically independent isolates were considered for this study, 45 from farms, 66 from slaughterhouses and 430 from retail.

Isolates were tested using the BD Phoenix automated system and the NMIC/ID-121 panels (BD, USA). Drugs included in the panel and the tested concentrations are available in S1 Table. Minimal Inhibitory concentrations (MIC) were interpreted using CLSI 2013 guidelines [28]. Strains resistant to cefotaxime (MIC ≥ 4 mg/L) were screened for the presence of ESBL/AmpC genes. Additional species confirmation was performed with matrix-assisted laser desorption ionization—time of flight mass spectrometry (MALDI-TOF MS) (Bruker, Delft, the Netherlands). The isolates originated from baseline studies that were financed for specific years only during the development of Coipars (Table 1). As a result for some years no samples were included.

**Table 1:** Distribution of cefotaxime-resistant *E*. *coli* in Colombian poultry (A) and ESBL/AmpC genes and isolates selected for plasmid and strain characterization (B).

	Source	Farms	Slaughter	Retail	Total
(A)	2008	8 / 29^a^	13 / 29	-	21 / 58
	2009	4 / 9	-	20 / 165	24 / 174
	2010	-	-	44 / 168	44 / 168
	2011	-	-	30 / 97	30 / 97
	2012	1 / 7	6 / 20	-	7 / 27
	2013	-	6 / 17	-	6 / 17
	2008–2013	13 / 45	25 / 66	94 / 430	132 / 541
(B)	*bla*_CMY-2_	8 (2)^b^	14 (6)	70 (13)	92 (21)
	*bla*_SHV-12_	2 (1)	5 (2)	7 (1)	14 (4)
	*bla*_SHV-5_	- (0)	1 (1)	2 (0)	3 (1)
	*bla*_CMY-2_-*bla*_SHV-12_^c^	-	1	3	4
	*bla*_CMY-2_-*bla*_SHV-5_	-	-	2	2
	*bla*_CTX-M-15_	-	-	1	1
	*bla*_CTX-M-2_	1	2	2	5
	*bla*_CTX-M-8_	-	1	-	1
	Total	11 (4)	24 (13)	87 (22)	122 (39)

^a^ Cefotaxime-resistant / Tested isolates

^b^ Numbers of isolates selected for plasmid and strain characterization are presented in brackets

^c^ All isolates positive for combinations of *bla*_CMY_-*bla*_SHV_ and *bla*_CTX-M_ were selected for plasmid and strain characterization

### ESBL/AmpC gene characterization

Polymerase chain reaction (PCR) with previously described primers was used to screen for ESBL [29] and plasmidic AmpC [30]. The genes analysed included *bla*_CTX-M_, *bla*_CMY_, *bla*_SHV_, *bla*_TEM_, *bla*_OXA-1-like_, *bla*_OXA-2-like_ and *bla*_OXA-10-like_. PCR products were purified using ExoSAP-IT according to manufacturer’s protocol (Affymetrix, USA) and sent to BaseClear (the Netherlands) for sequencing. Sequence results were analysed with BioNumerics v7.5 software (Applied Maths, Belgium) and compared with reference sequences deposited at http://lahey.org/studies/.

### Plasmid and strain characterization

A selection of strains based on the distribution of ESBL/AmpC variants was made and further characterized. Due to its low prevalence, the selection included all isolates positive for *bla*_CTX-M_ (n = 7) and all combinations of *bla*_CMY_-*bla*_SHV_ (n = 6). For the highly prevalent *bla*_CMY_ and *bla*_SHV_ genes, a random selection was made using the Random function in Microsoft^®^ Excel to assign random numbers to isolates positive for *bla*_CMY_ and *bla*_SHV_. The size of selection was fixed to a maximum of 30% for both genes. Finally, strains with the largest 21 and 5 numbers, representing 23% and 29% of the isolates, were selected for *bla*_CMY_ and *bla*_SHV_ respectively.

Plasmids were purified from the original donor strain using the Qiagen Plasmid Midi-kit (Qiagen, Germany). Plasmid DNA was transformed into ElectroMAX DH10B cells through electroporation (Invitrogen, USA) by mixing 5μl of isolated plasmid DNA with 20μl of electro-competent cells. Electroporation conditions were 25 μF, 200 Ω, and 2.0 Kv. Cells were recovered for 1 hour at 37°C in SOC-medium (Invitrogen, USA). Subsequently, transformants were selected on Luria-Bertani agar (LΒ-agar) supplemented with 2 mg/L cefotaxime (Sigma, USA). Conjugation experiments were performed when electroporation did not yield transformants. Donor strain and recipient rifampicin-resistant strain *E*. *coli* W3110, were grown separately overnight in 5 ml of LΒ-Broth. Volumes of 0.1 ml of donor and 0.9 ml of recipient strains were added to 9 ml of LB broth and cultured overnight. Conjugants were selected on LΒ-agar supplemented with 2 mg/L cefotaxime (Sigma, USA) and 75 mg/L rifampicin (Sigma, USA). Lysates of DNA for confirmation of ESBL/AmpC genes, PCR Based Replicon Typing (PBRT), plasmid Multi Locus Sequence Typing (pMLST), plasmid Double Locus Sequence Typing (pDLST) and plasmid Replicon Sequence Typing (pRST) were obtained from transformants and transconjugants. PBRT was performed using the PBRT-kit (Diatheva, Italy) following the manufacturer instructions. Noteworthy, the kit did not differentiate between IncI1 or IncIγ plasmids. For readability all IncI1-Iγ plasmids are here designated as IncI1. Plasmid MLST, pDLST and pRST were performed as previously described for IncI1, IncHI2 and IncF plasmids, respectively [31–33].

For Multi Locus Sequence Typing (MLST), bacterial lysates were obtained after boiling the original donor strain and PCR protocols were performed as previously described at http://mlst.warwick.ac.uk/mlst/dbs/Ecoli/. PCR products were purified and sequenced as described above.

Allele and profile analysis for pMLST, pDLST and pRST was done at http://pubmlst.org/plasmid/. Sequences and minimal spanning trees for MLST were analysed with BioNumerics v7.5 software (Applied Maths, Belgium).

## Results

### Screening of resistance, ESBL/AmpC genes and selection of isolates for molecular characterization

In total 132 of 541 strains were phenotypically resistant to cefotaxime (MIC≥ 4mg/L), 13 from farms, 25 from slaughterhouses and 94 from retail (Table 1). From these, 122 were positive for ESBL and/or AmpC genes, 11 from farms, 24 from slaughterhouses and 87 from retail (Table 1). The most prevalent gene variant at the farm, slaughterhouse and retail level was *bla*_CMY-2_, present in 73% (n = 8/11), 58% (n = 14/24) and 80% (n = 70/87), respectively. Second most prevalent was *bla*_SHV-12_, present in 18% (n = 2/11), 21% (n = 5/24) and 8% (n = 7/87), respectively (Table 1). In addition to these gene variants we found *bla*_SHV-5_ (n = 3), *bla*_CTX-M-15_ (n = 1), *bla*_CTX-M-2_ (n = 5), *bla*_CTX-M-8_ (n = 1) and combinations of *bla*_CMY-2_-*bla*_SHV-12_ (n = 4) and *bla*_CMY-2_-*bla*_SHV-5_ (n = 2) distributed in the three levels. After sequencing, all positive reactions for *bla*_TEM_ PCR, were identified as *bla*_TEM-1_ or *bla*_TEM-1b_ and were considered as wild-type beta-lactamase. None of the isolates were positive for *bla*_OXA-1-like_, _-2-like_ or _-10-like_ genes.

Based on the distribution of gene variants, 39 isolates were selected and further characterized at the plasmid level using PBRT, pMLST, pDLST and pRST (if applicable) and at the strain level using MLST. The selection was aimed to investigate the diversity of genetic determinants and cover as much plasmid and strain STs as possible. Accordingly, all strains positive for *bla*_CTX-M_ (n = 7) and all for *bla*_CMY_-*bla*_SHV_ (n = 6) were included to cover the diversity or homogeneity of plasmids and strains associated to their spread. In contrast, a random selection was performed for the strains positive for *bla*_CMY_ (n = 92) and *bla*_SHV_ (n = 17) (Table 1).

### Plasmid typing

**Fig 1:**
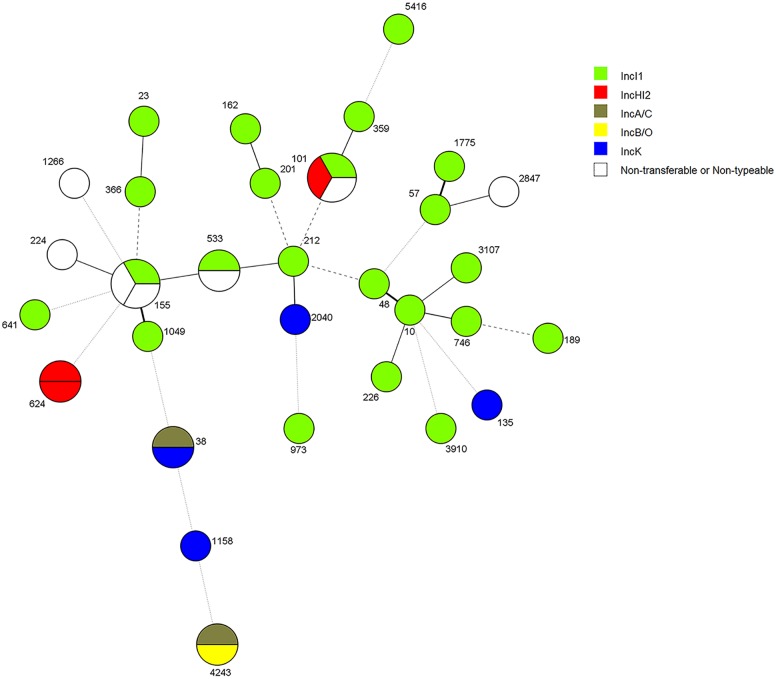
Distribution of plasmid families in ESBL/AmpC-producing *E*. *coli* from different Sequence Types in the Colombian poultry chain.

ESBL/AmpC genes from 36 strains were transferable using electroporation or conjugation. In the remaining 3 strains (EC1.6, EC10PP62457 and UGCAR489EC) the genes were non-transferable (Table 2). The transferred genes of 33 strains were located on a single plasmid replicon in the recipient strain after selective isolation. For 1 strain the plasmids remained non-typeable (Fig 1). IncI1 plasmids were most abundant in isolates from all years, sources and department of origin of the samples (22/33), except for the department of Bolivar (Table 2). IncK (4/33), IncHI2 (3/33), IncA/C (2/33), IncΒ/O (1/33) and a non-typeable replicon (strain FBOG54) were also associated with ESBL/AmpC-genes at a low frequency (Fig 1 and Table 2). Overall, IncI1 plasmids were associated with carriage of *bla*_CMY-2_, *bla*_SHV-12_ and *bla*_CTX-M-8_ genes, IncHI2 with carriage of *bla*_CTX-M-2_. Further plasmid typing, pMLST for IncI1 and pDLST for IncHI2, demonstrated IncI1/ST12 (13/22) and the new IncHI2/ST7 (2/3) as the most abundant plasmid Sequence Types, respectively (Table 2). In addition to IncHI2/ST7, two new IncI1 STs were observed, designated as ST229 and ST230 (Table 2). Three IncI1 and one IncHI2 plasmid remained non-typeable due to missing amplification of one of the alleles in the pMLST and pDLST schemes (Table 2). Information relating to the year, source and origin of the strains with their respective gene, ST, incompatibility group (PBRT) and plasmid ST is shown in Table 2.

**Table 2:** Strain and plasmid typing on selected strains after ESBL/AmpC genes characterization.

Source	Year	Location (Department)	Strain	ESBL/AmpC enzymes in original isolates^a^	Plasmid replicons	Plasmid ST/FAB formula	Strain ST	Clonal Complex
FARM	2008	Santander	ECSLV.10.C1	SHV-12	I1^b^	Non-typeable^e^	366	-
		Santander	ECSXXXIV.1.C	CMY-2	I1^b^	12	57	350
	2009	Cundinamarca	EC1.6	CTX-M-2	-	-	1266	-
		Cundinamarca	EC4.5	CMY-2	I1^b^	Non-typeable^e^	101	101
SLAUGHTER	2008	Santander	ECSIIIL.9.C1	CMY-2	I1-F^d^	12-F24:A-:B1	155	155
		Santander	ECSIIL.18.C2	SHV-12	I1^b^	12	3107	-
		Santander	ECSIVL.9	CTX-M-2	HI2^b^	Non-typeable^e^	101	101
		Santander	ECSLII.8.C3	CMY-2	I1^b^	12	155	155
		Santander	ECSLIV.27.C2	CMY-2	I1^b^	12	10	10
		Santander	ECSLIV.7.C1	CMY-2	I1^b^	Non-typeable^e^	3910	-
		Santander	ECSVIL.11.C1	CMY-2/SHV-12	I1^b^	12	359	-
		Santander	ECSVIL.21.C3	CMY-2	I1^b^	12	212	-
		Santander	ECSXXXII.7	CTX-M-2	A/C^b^		38	38
	2012	Cundinamarca	FBOG54	SHV-5	Non-typeable^b^		155	155
		Santander	FSAN126	CMY-2	I1^b^	231	1775	-
	2013	Sucre	FSUC314	CTX-M-8	I1^b^	114	641	86
		Magdalena	FMAG347	SHV-12	I1^c^	230 ^g^	226	226
RETAIL	2009	Cundinamarca	EC102.1	CMY-2	I1^c^	229 ^g^	23	23
		Cundinamarca	EC107.1	CTX-M-2	HI2^b^	7 ^g^	624	648
		Cundinamarca	EC108.1	CTX-M-2	HI2^b^	7 ^g^	624	648
		Cundinamarca	EC114.1	CMY-2/SHV-12	B/O^b^		4243	-
		Cundinamarca	EC10PP62457	CMY-2	-	-	101	101
		Cundinamarca	EC9PP62328	SHV-12	I1^b^	230 ^g^	973	-
	2010	Cundinamarca	UGBOG166EC	CMY-2	K^b^		2040	-
		Cundinamarca	UGBOG204EC	CMY-2/SHV-5	I1^b^	12	189	165
		Cundinamarca	UGBOG301EC	CMY-2	I1^b^	12	201	469
		Cundinamarca	UGBOG304EC	CMY-2/SHV-12	K^b^/ I1^b^^f^	26	746	-
		Cundinamarca	UGBOG34EC	CMY-2	I1^b^	12	48	10
		Meta	UGVIL369EC	CMY-2/SHV-12	I1^b^	12	1049	-
		Meta	UGVIL380EC	CMY-2	K^c^		135	-
	2011	Atlántico	UGBAR389EC	CMY-2	I1^b^	12	5416	-
		Atlántico	UGBAR425EC	CMY-2	I1^b^	12	533	-
		Atlántico	UGBAR428EC	CMY-2	I1-F^d^	12-F29:A-:B-	533	-
		Bolívar	UGCAR489EC	CTX-M-15	-	-	224	-
		Bolívar	UGCAR500EC	CMY-2	K^b^		1158	-
		Cundinamarca	UGCAR511EC	CMY-2	A/C^c^		4243	-
		Córdoba	UGMON457EC	CMY-2/SHV-5	I1^b^	12	162	469
		Boyacá	UGSAN546EC	CMY-2	I1-F^d^	Non-typeable^e^-F36:A1:B1	2847	-
		Boyacá	UGTUN878EC	CMY-2	K^b^		38	38

^a^ Underlined genes were transferable to recipient strains by electroporation or conjugation of plasmids

^b^ Single replicon transferred by electroporation or

^c^ by conjugation

^d^ Both replicons were transferred together by conjugation. It is uncertain in which of the plasmids the ESBL/AmpC gene was transferred

^e^ No PCR amplification product for one of the alleles

^f^ *bla*_CMY-2_ and *bla*_SHV-12_ were transferred in IncK and IncI1 plasmids respectively

^g^ New plasmid sequence types

### Strain typing

Thirty-one different Sequence Types (STs) were detected among the 39 strains selected (Fig 1 and Table 2). Sequence Types ST38 (n = 2), ST101 (n = 3), ST155 (n = 3), ST533 (n = 2), ST624 (n = 2) and ST4243 (n = 2) were found more than once. ST155 and ST4243 were found in slaughterhouses and retail, respectively. ST533 and ST624 were found both in retail but ST533 in 2011 and ST624 in 2009. ST38 was encountered in different years and sources. Finally, ST101 was found in samples from farms (n = 1), slaughterhouses (n = 1) and retail (n = 1) in different years.

## Discussion

We have provided the molecular characterization of ESBL/AmpC-producing *E*. *coli* from different base-line studies within the Colombian poultry production chain. Our selection aimed to reflect the characteristics of resistance in different Colombian departments in order to comply with the country-wide scope of an integrated program like Coipars. We have found that the most prevalent genes and plasmids were present in highly heterogeneous strain STs, which may well be indicative for non-clonal spread of resistance within the poultry chain. In addition, our results suggest that dissemination of the most prevalent genes, *bla*_CMY-2_ and *bla*_SHV-12_, is mainly through horizontal transfer of plasmids belonging to the IncI1 group.

Among the systems providing automation to the phenotypic screening of ESC resistance, Vitek 2 (bioMérieux) and BD Phoenix have been used in the investigation of ESBL-producing *E*. *coli* in animal isolates [34–37]. Although Vitek 2 is most commonly used, no dedicated assessment of their performance for isolates from animal origin is available in the literature. When testing human isolates in studies from Europe and the USA, Phoenix has been found to provide a better sensitivity and specifity in comparison with other commercial systems [38–41]. In our study, Phoenix was used for detection of resistance to cefotaxime regardless of its ESBL evaluation. In general, this drug is preferred among other cephalosporins given its advantage to cover both ESBL and plasmidic AmpC phenotypes [42]. In this respect, the automated phenotypic screening of cefotaxime resistance enabled the analysis of a large number of isolates with good discriminatory power. In total 122 out of 132 resistant strains were found to carry ESBL/AmpC genes, showing good performance of the automated system to screen large bacterial collections. The remaining isolates may be chromosomal *ampC*-promoter mutants or harbour a gene we did not screen for.

Our study has some limitations related to the limited number of isolates from farms and slaughterhouses from which a convenient selection of isolates was available. The exclusion of epidemiologically related isolates resulted in a marked reduction of samples, from 155 to 45 at farms and from 182 to 66 at slaughter. This exclusion may have influenced the prevalence of genes, plasmids and strains we encountered since it resulted in the uneven distribution of samples between the 3 levels of production. Additionally it may have resulted in loss of coverage of the diversity of genetic determinants within same production flocks. Nevertheless, this was a requisite to avoid general overrepresentation of genes, plasmids and strains due to sample duplicity. Thus, the characteristics of resistance were reported individually for the 3 different levels and our results served principally as a base line for the Colombian poultry chain. In addition, we included all isolates from retail and finally found that the proportion of the two most prevalent genes, *bla*_CMY-2_ and *bla*_SHV-12_, was equal in the three sources. This strongly suggests that in our collection of *E*. *coli* the distribution of ESBL/AmpC genes in the 3 levels of production we sampled are comparable.

### Gene frequencies

Other studies of ESBL/AmpC-producing *E*. *coli* from poultry in Latin America are scarce. However, available reports from Brazil have showed that together with *bla*_CMY-2_ [43], *bla*_CTX-M-2_ and *bla*_CTX-M-8_ are important mediators of ESC-resistance in *E*. *coli* from poultry and chicken meat [44–48]. It is important to note that some of these studies only screened for *bla*_CTX-M_ and did not include detection of plasmidic AmpC genes in their PCR screening. This will affect the accuracy of a comparison with the prevalences described in this study. Furthermore, studies from the USA and Canada demonstrated that resistance to third generation cephalosporins in samples from chicken meat is predominantly mediated by *bla*_CMY-2_ [49–54]. In our study, the prevalence of *bla*_CMY-2_ was higher than other genes within the three sources. We found a proportion of over 58% in ESBL/AmpC positive strains from farms, slaughter and retail. In our case, the encountered prevalence was based on random isolation of *E*. *coli* (that is, without selection using antimicrobials during enrichment). Therefore, the frequencies of other genes than *bla*_CMY-2_ may be underestimated and comparisons with reports using selective isolation with cephalosporins during enrichment have to be interpreted carefully. Our finding of this gene as the main mediator of ESC resistance, is in accordance with previous studies (using selective isolation) from European countries like Denmark, Sweden, and the Netherlands where *bla*_CMY-2_ was found to be highly prevalent at grandparent and parent level of the broiler production pyramid [55–57].

This is the first publication from Colombia in relation to ESBL/AmpC—producing *E coli* from poultry and chicken meat. In reports from human isolates, *bla*_CTX-M-12_, *bla*_CTX-M-15_, *bla*_SHV-12_ and *bla*_SHV-5_ [19–22] were most prevalent. More recently, *bla*_CMY-2_ has been included as part of the genotypic screening and associated to community onset infections caused by *E*. *coli* ST131 [23]. Although previous studies did not characterize the plasmids mediating the resistance, our findings suggest a possible relationship with some of the ESBL/AmpC genes found in humans in Colombia, namely *bla*_CMY-2_, *bla*_SHV-12_, *bla*_SHV-5_ and *bla*_CTX-M-15_. Further studies using a one health approach, simultaneous collection of samples from human and animal sources and sequence-based molecular characterization of plasmids and strains are necessary to confirm this relationship.

### ESBL/AmpC carrying plasmids

Various plasmid families, namely IncI1, IncK, IncF, IncHI2, IncA/C and IncΒ/O were found harbouring ESBL/AmpC genes. However, we have shown that the association of *bla*_CMY-2_ and *bla*_SHV-12_ was mainly to IncI1 plasmids. Both the diversity of the analysed strains (year, source and place of isolation) and the diversity in *E*. *coli* STs, in contrast to the homogeneity in plasmid types, support the hypothesis that spread of ESBL/AmpC in the Colombian poultry chain is mainly mediated by horizontal gene transfer of plasmids. A minimal spanning tree representing the genetic relatedness of *E*. *coli* STs and plasmid lineages is shown in Fig 1.

In contrast to strain Sequence Types, there is evidence that supports the association between specific plasmid Sequence Types and the spread of particular ESBL/AmpC genes. In a previous study, Smith and colleagues [58] reported the combination of IncI1/ST12 and *bla*_CMY-2_ in more than 80% of the plasmids they analysed. Among our isolates, *bla*_CMY-2_ was the most frequently encountered gene, and IncI1/ST12 was most frequently associated to its spread. In particular, we found 12 isolates carrying *bla*_CMY-2_ in IncI1/ST12 plasmids out of 16 carrying *bla*_CMY-2_ in overall IncI1 plasmids. It is likely that this combination of plasmid ST-gene is widespread in Colombian poultry since *bla*_CMY-2_ was the most prevalent gene in our study and IncI1 accounted for most of its occurrence (16/24). The features associated to this plasmid ST and its interactions with *E*. *coli* hosts are of further interest to assess the factors influencing the successful spread of *bla*_CMY-2_ in poultry.

### Sequence types of ESBL/AmpC producers

As a result of our selection, different samples from all years and levels of production were characterized. Some departments were represented with more than one sample, namely Cundinamarca (n = 15) Santander (12), Atlántico (3), Meta (2), Bolívar (2) and Boyacá (2). In addition single samples from Sucre, Magdalena and Córdoba were included. In general, we observed high heterogeneity of strain STs harbouring same combinations of gene variants and plasmids (Fig 1 and Table 2). In addition, but to a lesser extent, samples originating from the same years, levels of production and departments carried the same genes, plasmids and strain STs, as observed for ST155 (Santander), ST624 (Cundinamarca) and ST533 (Atlántico).

From a plasmid perspective, IncI1 harbouring *bla*_CMY_ was distributed among highly heterogeneous *E*. *coli* STs from different years, levels of production and departments. However, it was also present among the closely related *E*. *coli* ST155 and ST533, in both cases carrying *bla*_CMY-2_ on an IncI1/ST12 plasmid (Table 2). In this respect, we conclude our selection of strains was sufficient to detect inter-department diversity and intra-department diversity to a limited level, and also the likelihood that in one geographical area *E*. *coli* belonging to the same ST exist. Nonetheless, an in-depth analysis for the spread within departments was out of the scope of the present study. This would require a much more intensive sampling scheme.

At a broader scale, seven out of 31 STs have been previously reported as ESBL/AmpC producers in samples from both humans and poultry, namely ST10, ST23, ST38, ST48, ST57, ST155 and ST624 [11,14,59–64]. One has only been found as ESBL/AmpC producer in poultry, ST641 [11,60,65,66] and 3 in humans, ST101, ST162 and ST746 [63,67]. Additionally, ST224, ST359 and ST973 have been previously reported in other livestock and companion animals [65,68,69]. To our knowledge this is the first report in which ESBL/AmpC producers are associated with ST135, ST189, ST201, ST212, ST226, ST366, ST533, ST1049, ST1158, ST1266, ST1775, ST2040, ST2847, ST 3107, ST3910, ST4243 and ST5416. At the level of isolates from human clinical isolates in Colombia, only two strains belonging to ST38 coincide in our selection. Different than *bla*_CTX-M-15_ [24] our strains carried *bla*_CMY-2_ or *bla*_CTX-M-2_ and were not considered as an evident link for the transmission of resistance.

In conclusion, the molecular characterization of ESBL/AmpC-producing *E*. *coli* has identified the genetic determinants mediating the spread of resistance to ESC in Colombian poultry and chicken meat. The differences in distribution of genes, plasmids and strains between our study and other reports may be related to different practices of farming and supply of chickens and chicken meat in the country. Further characterization of ESBL/AmpC-producing *E*. *coli* from human and poultry sources in Colombia is necessary to understand the potential transmission of resistance determinants. This approach could be enhanced by the use of next generation sequencing of isolates and plasmids.

## Supporting Information

S1 Table Drugs and range of concentrations tested with the BD Phoenix panels NMIC-121. (XLSX)
